# Case–Control Study of Long COVID, Sapporo, Japan

**DOI:** 10.3201/eid2905.221349

**Published:** 2023-05

**Authors:** Toshiaki Asakura, Takashi Kimura, Isaku Kurotori, Katabami Kenichi, Miyuki Hori, Mariko Hosogawa, Masayuki Saijo, Kaori Nakanishi, Hiroyasu Iso, Akiko Tamakoshi

**Affiliations:** Hokkaido University, Sapporo, Japan (T. Asakura, T. Kimura, I. Kurotori, K. Kenichi, A. Tamakoshi);; National Center for Global Health and Medicine, Tokyo, Japan (M. Hori, M. Hosogawa, H. Iso);; Health and Welfare Bureau, Sapporo (M. Saijo, K. Nakanishi)

**Keywords:** COVID-19, respiratory infections, severe acute respiratory syndrome coronavirus 2, SARS-CoV-2, SARS, coronavirus disease, zoonoses, viruses, coronavirus, long COVID, post–COVID-19 condition, Japan

## Abstract

We conducted a cross-sectional survey among SARS-CoV-2–positive persons and negative controls in Sapporo, Japan, to clarify symptoms of long COVID. We collected responses from 8,018 participants, 3,694 case-patients and 3,672 controls. We calculated symptom prevalence for case-patients at 2–3, 4–6, 7–9, 10–12, and 13–18 months after illness onset. We used logistic regression, adjusted for age and sex, to estimate the odds ratio (OR) for each symptom and control reference. We calculated symptom prevalence by stratifying for disease severity, age, and sex. At 4–18 months from illness onset, ORs for anosmia, ageusia, dyspnea, alopecia, and brain fog were consistently >1, whereas ORs for common cold–like, gastrointestinal, and dermatologic symptoms were <1. Time trend ORs increased for diminished ability to concentrate, brain fog, sleep disturbance, eye symptoms, and tinnitus. Clinicians should focus on systemic, respiratory, and neuropsychiatric symptoms among long COVID patients.

Several months after the COVID-19 pandemic began, patients coined the term long COVID to describe the fluctuated, progressive, persistent, and multiphasic symptoms caused by SARS-CoV-2 infection (*1*). This patient-derived notion was rapidly adopted, and many biomedical research studies were conducted using a variety of definitions and terms, such as post–COVID-19 syndrome and post-acute sequelae of SARS-CoV-2 infection (*2*).

The World Health Organization (WHO) conducted a Delphi process to create a consensus definition of post–COVID-19 condition for adult patients (*3*). The WHO consensus definition states that “post–COVID-19 condition occurs in individuals with a history of probable or confirmed SARS-CoV-2 infection, usually 3 months from the onset of COVID-19, with symptoms that last for at least 2 months and cannot be explained by an alternative diagnosis” (*3*). The WHO consensus definition also pointed out that common symptoms of post–COVID-19 condition were fatigue, shortness of breath, and cognitive dysfunction and that these symptoms affected everyday functioning among patients.

Although symptom prevalences among COVID-19 patients are reportedly high, demonstrating that such prevalences are specific to COVID-19 patients versus uninfected persons has been difficult, and only a few cohort studies have been conducted with controls (*4*,*5*). One national cohort study reported symptom prevalence among children and adolescents 3 months after SARS-CoV-2 test-positive date and among test-negative controls (*5*). The study showed that the number of symptom types in case-patients was higher 3 months after the test-positive date than at the time they received a PCR test. That study also showed that COVID-19 case-patients generally had more symptom types than the control group (*5*), emphasizing the need for controls to accurately assess long COVID. However, that study did not assess the 6-month or 12-month effects of SARS-CoV-2 infection on health and focused only on children. Another study used a small sample size and did not comprehensively investigate symptom characteristics (*4*). Another approach to set controls is by using electronic health records (*6*,*7*). The Centers for Disease Control and Prevention reported that COVID-19 potentially had long-term effects on multiple organs and caused a wide range of diseases (*6*). In a study in which patients with acute respiratory infection other than COVID-19 were set as controls, prevalence of neurologic complications was substantially higher among COVID-19 case-patients than controls (*7*). However, those studies did not have consistent symptomatic information and only assessed diagnosed diseases. Therefore, symptomatic characteristics of long COVID could not be explored by those approaches.

The complicated sequelae, long-term health effects, and health outcomes among adult COVID-19 patients remain unknown. To clarify the clinical features and long-lasting manifestations of SARS-CoV-2 infection, we conducted a large case–control cohort study in Sapporo, Japan, during the beginning of the first Omicron wave in February 2022. We analyzed cross-sectional baseline data to distinguish COVID-19–related long-term symptoms from non–COVID-19 symptoms by setting controls and using the WHO definition of post–COVID-19 condition (*3*).

## Methods

### Study Design and Participants

**Figure 1:**
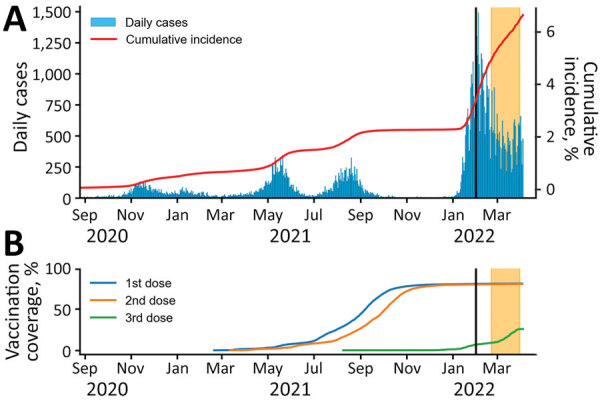
Daily COVID-19 cases, cumulative incidence, and vaccination rates during a case–control study of long COVID, Sapporo, Japan, August 2020–March 2022. A) Number of daily COVID-19 cases and cumulative incidence rates. B) Vaccination rates among the population of Sapporo. Date range represents timeframe in which participating case-patients might have been infected. Vertical black lines indicate February 1, 2022, the date potential participants were randomly identified. Yellow shading at right indicates the period of the survey in this study, February 21–March 31, 2022.

We collected cross-sectional data of COVID-19 patients and a control group in Sapporo, Japan, in a cohort study of long-term health effects of SARS-CoV-2 infection among adults. We randomly selected laboratory-confirmed COVID-19 case-patients and controls from among residents 20–64 years of age in Sapporo on February 1, 2022. We selected case-patients from the registry of the Sapporo Public Health Office, regardless of which SARS-CoV-2 variants were circulating at the time of their infections. Controls were persons who did not have a COVID-19 diagnosis as of February 1, 2022; we randomly sampled the control group to match the age and sex distribution of the general population of Sapporo. We mailed information about the study and the protocol to the selected possible participants, who then accessed and answered web-based questionnaires by using a URL provided (Appendix). We assessed the COVID-19 epidemiologic situation in Sapporo from daily cases, cumulative incidence, and vaccination coverage (Figure 1).

### Definition of Symptoms and Data Collection

To define case-patient symptoms, we used the WHO definition for post–COVID-19 condition (*3*), which considers a long COVID symptom as any symptom that developed after the initial illness onset, lasted for at least 2 months, and could not be explained by an alternative diagnosis. Using this definition, we asked case-patients about each symptom at the time they answered the questionnaire and at various timepoints after illness onset: 2–3, 4–6, 7–9, and 10–12 months. Although symptoms 2–3 months after illness onset were not exactly matched with the post–COVID-19 condition definition, we used the 2–3-month period as a proxy for relatively short-term influences of SARS-CoV-2 infection on the development and persistence of symptoms. We asked the control group about symptoms lasting for at least 2 months at the time they responded to the questionnaire. Of note, we only applied the WHO definition of long COVID symptoms to case-patients. All participants were asked about 31 symptom types, including COVID-19–related symptoms such as fatigue, ageusia, and anosmia (*8*–*10*) and common symptoms like headache, constipation, and diarrhea.

We mapped the symptoms to the Human Phenotype Ontology (HPO) terminology for consistency of research on long COVID (*11*). However, we did not follow HPO for 3 symptom types: tingling, abnormal menstruation, and eye symptoms. Tingling could be mapped to paresthesia, but the HPO term also included pricking or numbness of the skin, which is a broader meaning than we used. We asked about abnormal menstruation but did not include more granular HPO terminology, such as amenorrhea, irregular menstruation, and dysmenorrhea. In contrast, we asked about eye symptoms using a broader meaning than the prepared terminologies in the HPO; for eye symptoms, we included ocular pain, pruritus, gritty eye, hyperemia, epiphora, and blurred vision.

We collected age and sex information through the questionnaires and collected unique identifiers for patient data that were linked with the COVID-19 registry database operated by the Sapporo Public Health Office. We extracted information on test-positive date and acute-phase COVID-19 severity from the registry data, which were updated on April 5, 2022. Official documentation from the Ministry of Health, Labour and Welfare in Japan described classification of the severity (*12*); severity levels of moderate and severe were registered when COVID-19 patients were hospitalized. The National Vaccine Record System also provided vaccination status of participants registered in the database as of April 5, 2022.

The Sapporo Public Health Office anonymized all data and we used linkage keys to merge each dataset: our survey data, the COVID-19 registry from the Public Health Office, and the National Vaccine Registry. We made certain participants who received study information by mail could not be identified as COVID-19 cases by others. Participants received no compensation for answering the questionnaire. The study was approved by the ethical review board for Life Science and Medical Research, Hokkaido University Hospital, on February 10, 2022, under protocol code 021-0190.

### Statistical Analyses

Because some participants only answered for current symptoms, we mapped current symptoms to the appropriate time category based on the elapsed time from illness onset to response date. If onset date was missing, we imputed test-positive date subtracted by the mean incubation period for different SARS-CoV-2 variants: 7 days for wild-type, 5 days for Alpha variant, 4 days for Delta variant, and 3 days for Omicron (*13*). We assumed the wild-type SARS-CoV-2 variant for participants who tested positive through February 2021, Alpha variant for those who tested positive during March–June 2021, Delta variant for those who tested positive during July–December 2021, and Omicron variant for those who tested positive during January 2022. In addition, we created a 13–18-month category, which comprised only case-patients who had symptoms at the time of answering the questionnaire and whose illness onset was 13–18 months prior. We considered 1 month to be 30 days. In the questionnaire, we first asked whether the participant had symptoms lasting >2 months and then asked the timeframe in which they had each symptom they selected. For analysis of each symptom, we excluded participants who did not respond about the timeframe for each symptom.

We calculated symptom prevalences at each timepoint among case-patients and at the time of answering among controls. If the elapsed time from onset to answer date was shorter than each time category, we excluded those participants from the denominator. We used the Wilson score interval to calculate CIs (*14*). We also calculated stratified symptom prevalence of case-patients and controls stratified by illness severity, age, and sex. We applied logistic regression to calculate odds ratios of having symptoms among case-patients compared with controls and adjusted for age and sex. To assess co-occurrence of symptoms, we calculated co-occurrence matrices for symptoms and visualized their networks by using the Python package of NetworkX (https://networkx.org). 

We performed all analyses in Python version 3.9 (Python Software Foundation, https://www.python.org). We used the following packages for analyses: pandas version 1.4.1 (https://pandas.pydata.org) for data cleaning, matplotlib version 3.5.1 (https://matplotlib.org) for data visualization, Statsmodels version 0.13.2 (*15*) for calculation of the Wilson score interval and adjusted odds ratios, and NetworkX version 2.7.1 (*16*) for visualization of co-occurrence matrices.

## Results

### Participant Demographics

**Figure 2:**
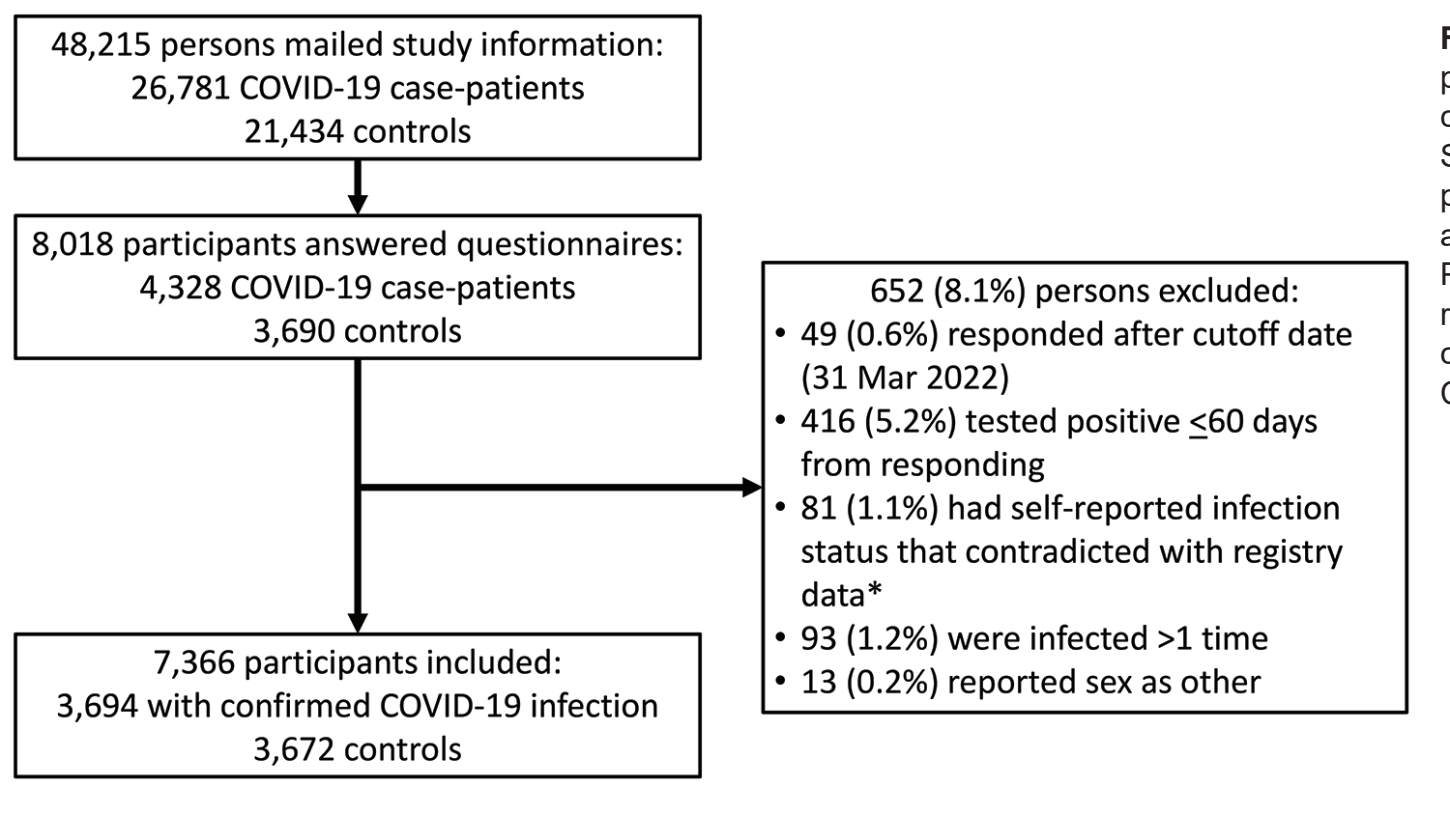
Flow chart of participant selection for a case–control study of long COVID, Sapporo, Japan. Controls were persons who self-reported as uninfected. *The Sapporo Public Health Office kept a registry of all the confirmed cases from the beginning of the COVID-19 epidemic.

We mailed study information to 26,781 possible case-patients and 21,434 possible controls. In all, 8,018 participants answered questionnaires. Case-patients included persons who were confirmed to be registered only once in Sapporo’s registry database on SARS-CoV-2 infections (Figure 2). Forty-seven participants had COVID-19 confirmed after January 1, 2022; 52 participants originally selected for the control group on February 1 answered as cases and had infections confirmed before questionnaires were sent in March. We used self-reported answers for age and included 93 participants who were 65 years of age, despite our original cutoff of 64 years.

Among participants, a higher percentage of persons 20–29 years of age (16.5%, 608/3,694) were among cases than among controls (11.4%, 420/3,672), whereas fewer persons 50–65 years of age (35.3%, 1,303/3,694) were among cases than among controls (41.3%, 1,516/3,672) (Table 1). The National Vaccine Record System provided the number of COVID-19 vaccinations as of April 5, 2022; the percentage of case-patients receiving no vaccination was nearly double that of the control group (11.0% vs. 6.0%). Among case-patients, 30.2% (1,117/3,694) reported symptoms at elapsed timepoints of 7–9 months, 34.2% (1,264/3,694) at 10–12 months, and 30.6% (1,132/3,694) at >13 months. For severity, 10.0% (370/3,694) of case-patients had a moderate or severe COVID-19 clinical course.

**Table 1:** Demographic of participants in a case–control study of long COVID, Sapporo, Japan*

Characteristics	No. (%)	
	Cases, n = 3,694	Controls, n = 3,672
Age group, y		
20–29	608 (16.5)	420 (11.4)
30–39	772 (20.9)	735 (20.0)
40–49	1,011 (27.4)	1,001 (27.3)
50–65	1,303 (35.3)	1,516 (41.3)
Sex		
M	1,528 (41.4)	1,591 (43.3)
F	2,166 (58.6)	2,081 (56.7)
No. COVID-19 vaccines†		
0	407 (11.0)	220 (6.0)
1	65 (1.8)	22 (0.6)
2	2,233 (60.4)	1,982 (54.0)
3	989 (26.8)	1,448 (39.4)
Time after illness onset, mo		
2–3‡	55 (1.5)	NA
4–6	126 (3.4)	NA
7–9	1,117 (30.2)	NA
10–12	1,264 (34.2)	NA
13–18	451 (12.2)	NA
>19	681 (18.4)	NA
Illness severity‡		
Asymptomatic	115 (3.1)	NA
Mild	2,952 (79.9)	NA
Moderate	334 (9.0)	NA
Severe	36 (1.0)	NA
Missing	257 (7.0)	NA

*NA, not applicable. †As of April 5, 2022. ‡Severity during acute phase of infection.

### Symptom Types at Designated Timepoint from Onset

**Figure 3:**
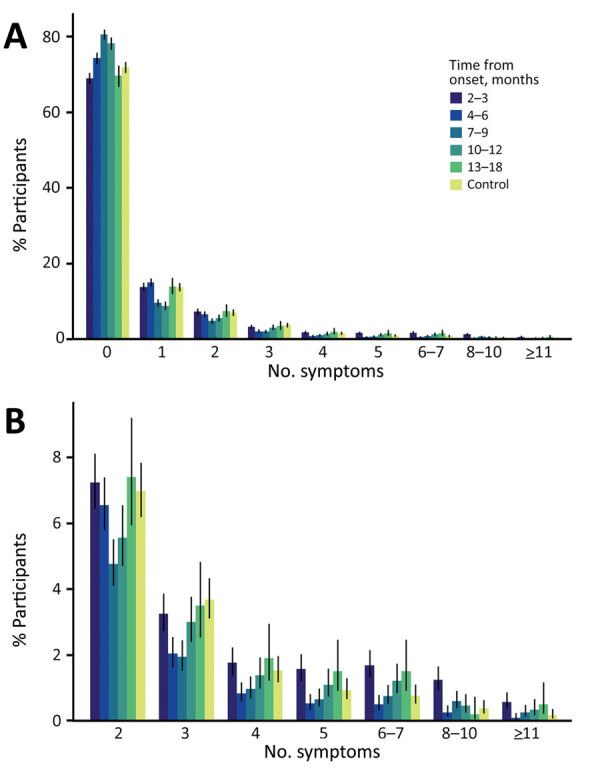
Percentage of symptoms reported among participants in a case–control study of long COVID, Sapporo, Japan. A) Number of symptoms at designated timepoints from onset among case-patients and among controls at the time of answering the questionnaire. B) Detail of percentage of participants with >2 symptom types. Error bars represent 95% CIs.

We calculated the number of symptom types observed at the designated timepoints for case-patients and at the time of questionnaire response for controls. We used 29 of 31 symptom types (Figure 3); we excluded erectile dysfunction and abnormal menstruation because those symptoms were sex-specific. Among case-patients, 31.1% (1,148/3,694) had >1 symptom at 2–3 months after illness onset, which is nearly the same percentage as reported symptoms at 13–18 months (30.5%, 305/1,001). When we focused on timepoints of 2–3 and 13–18 months, case-patients were likely to have more varieties of symptoms than controls; this tendency was more apparent in case-patient who had >5 symptom types.

### Prevalence of Each Symptom at Designated Timepoints 

**Figure 4:**
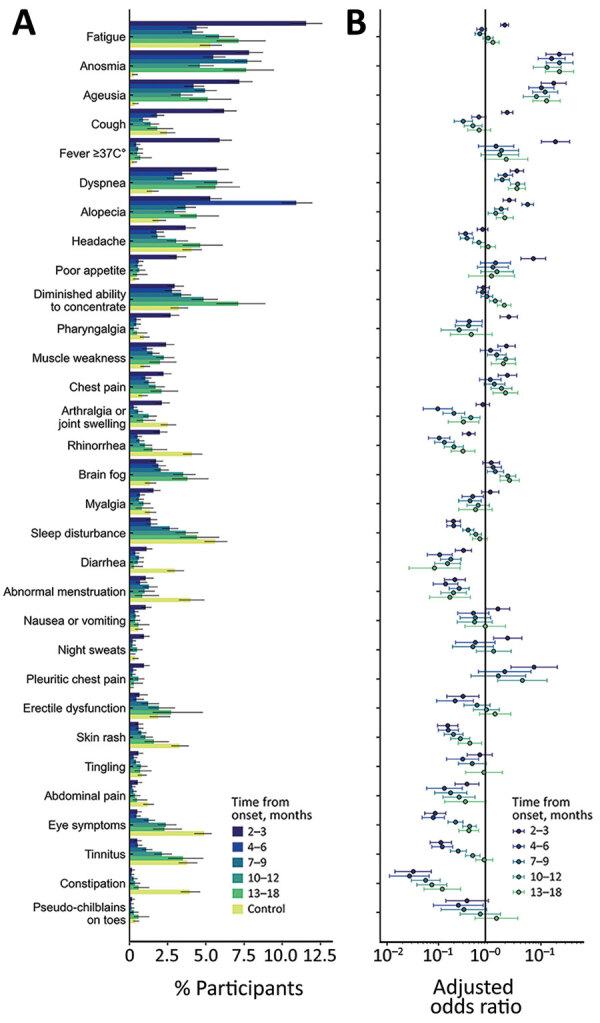
Prevalences of each symptom of cases at designated elapsed timepoints from onset including those of each symptom at the time of answering for controls (A), and adjusted odds ratios (B) in a case–control study of long COVID, Sapporo, Japan. Reference for the regression is based on controls. The order of symptoms described is listed in descending order in terms of the prevalence of symptoms of cases at 2–3 months after onset. Age and sex are adjusted. Some odds ratios for night sweats, pleuritic chest pain, and tingling are not displayed because of nonapplicability of the regression. The definition of symptoms, which developed and persisted after onset of COVID-19 and cannot be explained by an alternative diagnosis, is only applicable to cases.

We calculated the prevalence and adjusted odds ratio (aOR) of each symptom at designated timepoints for cases and at the time of response for controls (Figure 4; Appendix Tables 1, 2). Among all symptoms observed, fatigue accounted for the highest percentage, 11.55% (423/3,661), at 2–3 months after onset (aOR 2.36, 95% CI 1.97–2.81), and remained higher after 13 months (aOR 1.38, 95% CI 1.04–1.84).

Symptoms with an aOR >1 during all timepoints were anosmia, ageusia, dyspnea, and alopecia. Muscle weakness, chest pain, and brain fog had aORs >1 at 3 or 4 timepoints. Symptoms with explicitly higher aORs at 2–3 months than at other timepoints were fatigue, cough, fever, poor appetite, pharyngalgia, nausea or vomiting, and night sweats. We observed time trends of increasing aORs for diminished ability to concentrate, brain fog, sleep disturbance, tinnitus, and eye symptoms. Arthralgia or joint swelling, rhinorrhea, diarrhea, abnormal menstruation, skin rash, eye symptoms, and constipation had aORs <1 at all timepoints.

### Prevalences of Symptoms Stratified by Illness Severity, Age, and Sex

The prevalence of each symptom was more strongly related to disease severity than to age or sex (Appendix Figure 1). Prevalences for fatigue, dyspnea, alopecia, headache, diminished ability to concentrate, muscle weakness, chest pain, brain fog, myalgia, sleep disturbance, and nausea were higher among participants with moderate or severe COVID-19 cases than among those with asymptomatic and mild cases.

Case-patients >40 years of age had a higher prevalences of fatigue, dyspnea, alopecia, and diminished ability to concentrate (Appendix Figures 2, 3). Prevalences of fatigue and diminished ability to concentrate were lower among case-patients 20–29 years of age than among controls at 4–12 months from onset. Case-patients 30–65 years of age had higher prevalences of alopecia than did case-patients <30 years of age or controls.

Women had higher prevalences of anosmia, ageusia, and alopecia (Appendix Figure 4). The prevalence of anosmia for women at 13–18 months was nearly triple that for men. We observed a similar trend in ageusia, and higher proportions of women in both the case and control groups had alopecia. Although we performed comparisons within each sex, the prevalences of alopecia for male and female case-patients were consistently higher than those for controls.

### Co-occurrence Network of Symptom Types

**Figure 5:**
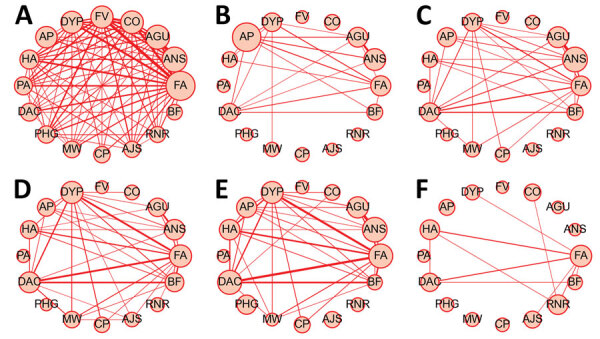
Co-occurrence network of symptoms among case-patients and controls in a case–control study of long COVID, Sapporo, Japan. A–E) Patients experiencing symptoms were queried at various timepoints after illness onset 2–3 mo (A), 4–6 mo (B), 7–9 mo (C), 10–12 mo (D), and 13–18 mo (E). F) Controls. Circle size and edge width are weighted based on number of occurrences; no edge indicates occurrence <0.5% of eligible participants. Counterclockwise order is based on the prevalence of each symptom at 2–3 months; we included the top 16 symptoms prevalent at 2–3 months from COVID-19 diagnosis. AGU, ageusia; AJS, arthralgia or joint swelling; ANS, anosmia; AP, alopecia; BF, brain fog; CO, cough; CP, chest pain; DAC, diminished ability to concentrate; DYP, dyspnea; FA, fatigue; FV, fever (temperature >37°C); HA, headache; MW, muscle weakness; PA, poor appetite; PHG, pharyngalgia; RNR, rhinorrhea.

We visualized co-occurrence networks and heatmaps of symptoms at each timepoint (Figure 5; Appendix Figure 5). Symptom co-occurrence at 2–3 months was more densely connected among case-patients than among controls. Among case-patients, fatigue mainly co-occurred with 15 other symptoms at 2–3 months. Dyspnea mainly co-occurred with fatigue, alopecia, and diminished ability to concentrate and weakly occurred with ageusia, muscle weakness, and chest pain. Also, anosmia and ageusia frequently co-occurred at each timepoint. Fatigue, alopecia, and diminished ability to concentrate occurred simultaneously among controls. Brain fog was related to diminished ability to concentrate and fatigue, but those relationships were more apparent among case-patients at 13–18 months after onset than at other timepoints.

## Discussion

We conducted a large cross-sectional study on prevalences of and risks for COVID-19 sequelae over multiple timepoints among the general population in Sapporo, Japan. The study revealed COVID-19–related symptoms that remained long after illness onset mainly were systemic, neuropsychiatric, or in the respiratory system (Table 2). Moreover, COVID-19 case-patients had a higher risk for ageusia, anosmia, muscle weakness, chest pain, poor appetite, and alopecia compared with controls. On the other hand, case-patients had the same or lower risk for several manifestations in musculoskeletal, gastrointestinal, and dermatologic systems compared with controls, suggesting weak or least likely long-term effects on these systems.

**Table 2:** Summarized adjusted odds ratio for each symptom in a case–control study of long COVID, Sapporo, Japan*

Symptoms	Adjusted odds ratio, by time after illness onset					Likeliness
	2–3 mo	4–6 mo	7–9 mo	10–12 mo	13–18 mo	
Systemic						
Fatigue	**>1.5**	<1	<1	>1	**>1**	Likely
Fever, temp. >37C°	**>1.5**	>1.5	>1.5	>1.5	>1.5	Likely
Headache	<1	<0.66	<0.66	<1	>1	Less likely
Night sweats	**>1.5**	<0.66	<0.66	>1	NA	Less likely
Respiratory						
Cough	**>1.5**	<1	<0.66	<0.66	<1	Unlikely
Dyspnea	**>1.5**	**>1.5**	**>1.5**	**>1.5**	**>1.5**	Very likely
Pleuritic chest pain	**>1.5**	>1.5	>1.5	**>1.5**	NA	Very likely
Neuropsychiatric						
Diminished ability to concentrate	<1	<1	>1	**>1.5**	**>1.5**	Likely
Brain fog	>1	>1	**>1.5**	**>1.5**	**>1.5**	Very likely
Sleep disturbance	<0.66	<0.66	<0.66	<0.66	<1	Unlikely
Otorhinolaryngological						
Ageusia	**>1.5**	**>1.5**	**>1.5**	**>1.5**	**>1.5**	Very likely
Anosmia	**>1.5**	**>1.5**	**>1.5**	**>1.5**	**>1.5**	Very likely
Pharyngalgia	**>1.5**	<0.66	<0.66	<0.66	<0.66	Unlikely
Rhinorrhea	<0.66	<0.66	<0.66	<0.66	<0.66	Unlikely
Tinnitus	<0.66	<0.66	<0.66	<0.66	<1	Unlikely
Musculoskeletal						
Muscle weakness	**>1.5**	>1	**>1.5**	**>1.5**	**>1.5**	Very likely
Arthralgia or joint swelling	<1	<0.66	<0.66	<0.66	<0.66	Unlikely
Myalgia	>1	<0.66	<0.66	<1	<0.66	Unlikely
Cardiovascular						
Chest pain	**>1.5**	>1	>1	**>1.5**	**>1.5**	Very likely
Gastrointestinal						
Poor appetite	**>1.5**	>1.5	>1	>1.5	>1	Likely
Diarrhea	<0.66	<0.66	<0.66	<0.66	<0.66	Unlikely
Nausea or vomiting	**>1.5**	<0.66	<0.66	<0.66	<1	Unlikely
Abdominal pain	<0.66	<0.66	<0.66	<0.66	<0.66	Unlikely
Constipation	<0.66	<0.66	<0.66	<0.66	<0.66	Unlikely
Dermatologic						
Alopecia	**>1.5**	**>1.5**	**>1.5**	**>1.5**	**>1.5**	Very likely
Skin rash	<0.66	<0.66	<0.66	<0.66	<0.66	Unlikely
Tingling	<1	<0.66	<0.66	NA	<1	Unlikely
Pseudo-chilblains on toes	<0.66	<0.66	<0.66	<1	>1.5	Less likely
Other						
Abnormal menstruation	<0.66	<0.66	<0.66	<0.66	<0.66	Unlikely
Erectile dysfunction	<0.66	<0.66	<1	>1	>1.5	Less likely
Eye symptoms	<0.66	<0.66	<0.66	<0.66	<0.66	Unlikely

*Reference for regression is based on controls. Bold text indicates 95% CI that does not contain 1. NA, not applicable.

COVID-19 case-patients had a wider variety of symptom types at 2–3 months and 13–18 months than did controls; the difference in the number of symptom types was more apparent in COVID-19 case-patients who had >5 symptom types, which is consistent with a previous study (*5*). In addition, we analyzed the time trend of aORs and cluster characteristics between symptoms for cases compared with controls (Figures 4, 5) The odds ratios for neuropsychiatric symptoms (diminished ability to concentrate, brain fog, and sleep disturbance) increased over elapsed timepoints, even after 13 months. On the other hand, typical common cold–like symptoms (cough, pharyngalgia, and rhinorrhea) disappeared 4 months after onset, although those prevalences were higher at 2–3 months among cases compared with controls. We noted cluster characteristics of symptoms among cases, especially between fatigue, dyspnea, alopecia, diminished ability to concentrate, and brain fog, but we observed similar cluster characteristics among controls.

COVID-19 is a systemic disease with diverse manifestations, which complicates the exploration of long COVID. A previous study in which COVID-19 cases were compared with matched controls by using electronic health records showed an increased risk for disease in an extensive range of organs and tissue types, including gastrointestinal organs, endocrine system, and renal organs, and the increased risks were observed even in analysis among a limited number of persons 18–64 years of age (*6*). Because that study used electronic health records for both cases and controls, results might be biased toward cases. In this study, we investigated general symptoms of COVID-19 cases and observed high odds ratios for systemic, respiratory, and neuropsychiatric symptoms. Potential mechanisms for differences in observed risks between the previous electronic health record–based studies and this study could be less severe hyperinflammatory status caused by infection (*17*). SARS-CoV-2 infection causes hyperinflammation, including cytokine storm, inducing production of endogenous chemical substances, and prothrombotic condition, which causes respiratory failure, pulmonary embolism, diarrhea, gastrointestinal hemorrhage, myocardial injury, and other systemic syndromes (*18*). However, cases with mild symptoms would not experience the severe hyperinflammatory status.

A prospective study on 15-year follow-up of patients with SARS (severe acute respiratory syndrome), a disease similar to COVID-19, showed long-term effects on pulmonary function, bone health, and lipid metabolism (*19*). Similar results were reported in an examination of SARS cases at 12 years after disease onset (*20*). Similarly, COVID-19 could affect long-term pulmonary function. Our study results showed prolonged effects on the respiratory system (dyspnea and pleuritic chest pain), even in mildly symptomatic cases, and a much higher prevalence of dyspnea and cough among severe cases, necessitating long-term and continuous monitoring of COVID-19 patients.

Other reports showed COVID-19 patients had high prevalences of neurologic complications several months after disease onset (*7*,*21*). Neuroinvasiveness and neurovirulence of SARS-CoV-2 are potential mechanisms of those neurologic complications (*22*). Our study demonstrated that mildly symptomatic cases had a high risk for neuropsychiatric symptoms, such as diminished ability to concentrate and brain fog. Those data suggest that immune-mediated damage, neurotropism, or neurovirulence properties of SARS-CoV-2 are involved in long-term effects. Those properties also could be related to autoimmune diseases and alopecia (*23*–*25*).

The prevalence of all symptoms reported in our study was lower than those reported through meta-analysis of cross-sectional studies (*10*,*26*). This difference might be a result of the difference in the definition of symptom. Our definition included conditions lasting at least 2 months, but other studies included only symptoms at the time of answering questions without asking how long symptoms had persisted. Furthermore, the difference in the definitions used between cases and controls in the current study caused the odds ratios of some symptoms to be <1.

 The first limitation of our study is that the results could be affected by recall bias, which is indicated by U-shaped prevalences of 22 of 31 symptom types. This bias might be explained by the evidence that 30.6% of case-patients reported symptoms >13 months after illness onset, and from evidence that 38.2% of case-patients who reported having that symptom only at the time of responding to the questionnaire. Those biases could introduce an underestimation of the prevalence of symptoms in case-patients at each elapsed timepoint. Second, the data obtained in this study were collected by self-reporting method, and patients might not have been able to decide whether their symptoms were related to their COVID-19 diagnosis or another illness. Third, we did not include vaccination effects in this study. Several studies showed the effectiveness of vaccination against long COVID (*27*,*28*), but in our study only a small number of case-patients were vaccinated >2 times before COVID-19 diagnosis, suggesting that the results obtained in this study were not largely biased by vaccination effect. Fourth, we compared long-term symptoms of case-patients with those of controls sampled from the general population of Sapporo, and that comparison might be biased if persons with certain demographic characteristics were more likely to be infected. Therefore, prevalences of symptoms for case-patients might have been higher than those of controls because of the difference in demographic characteristics of the population, not because of having SARS-CoV-2 infection.

In conclusion, among symptomatic case-patients, fatigue, dyspnea, and neuropsychiatric symptoms were key characteristics of COVID-19 sequelae over time, but most common cold–like, gastrointestinal, and dermatologic symptoms disappeared several months after illness onset. Clinicians evaluating patients for potential long COVID should focus on systemic, respiratory, and neuropsychiatric symptoms associated with long-term sequelae of severe COVID-19 and on ageusia, anosmia, and alopecia for patients who had mildly symptomatic cases.

Appendix Additional information on a case-control study of patients with post–COVID-19 condition, Sapporo, Japan.
