# Supplementary material for: Case–Control Study of Long COVID, Sapporo, Japan
Source: Emerg Infect Dis. 2023 May;29(5):956–66. doi: 10.3201/eid2905.221349 (PMC10124665; doi:10.3201/eid2905.221349)
Supplement: Appendix — Additional information on a case-control study of patients with post–COVID-19 condition, Sapporo, Japan. [file 22-1349-Techapp-s1.pdf]

Article DOI: https://doi.org/10.3201/eid2905.221349

Case–Control Study of Long COVID, Sapporo, Japan

Appendix

Details of the Cohort Study

The Department of Public Health, Hokkaido University launched the cohort study on the long-term health impacts of SARS-CoV-2 infections among adults aged 20 to 64 in Sapporo, Japan. In this study, we recruited participants from the laboratory-confirmed COVID-19 cases and the controls who were not registered in Sapporo's registry.

The cases were randomly sampled from the available data in the registry, which recorded all the confirmed patients in Sapporo from the beginning of the COVID-19 epidemic. Controls were randomly selected from residents in Sapporo who had never been diagnosed as COVID-19–positive to match the age and sex distribution of the general population in Sapporo. The selection of cases and controls was conducted on February 1, 2022, on which the cumulative number of cases aged 20 to 64 was 39,729. In the end, we sent questionnaires to 54.0% (21,434/39,729) of cases.

The documents, in which the purposes and the protocol of the present study as well as unique individual URLs for access to the web-based questionnaires were written, were sent to each selected participant by mail. If participants agreed with the purposes and the protocol of the present study, they accessed the web-based questionnaires and answered questions. We sent the documents to participants on February 21, 2022, and March 3, 2022, and opened the server on February 21, 2022, which was planned to be operated by March 31, 2022.

The participants were first asked about their history of infection and based on this answer, they proceeded to the corresponding questionnaires for cases and controls. The main differences in questionnaires were questions for the time course of 31 general symptoms considered to be related to long-term sequelae of COVID-19, which are the main outcomes of the present study. Cases were asked to "Choose all the symptoms lasting for at least 2 months

which can not be explained by the alternative diagnosed diseases and developed after SARS-CoV-2 infection," and if they answered "yes" for each symptom, they were further required to answer the existence of each symptom during each period from their initial onset. Periods for cases were "until 3 months," "4 to 6 months," "7 to 9 months," or/and "10 to 12 months" from onset as well as the present symptoms. Therefore, participants can report fluctuations in their symptoms. For example, participants could only answer for "4 months to 6 months" and "10 months to 12 months." Some participants did not answer any period even though they answered yes to the existence of a symptom. Controls were asked to "Choose all the present symptoms lasting for at least 2 months," and if they answered "yes" for each symptom, controls further chose "before the COVID-19 pandemic" or "after the COVID-19 pandemic."

Sapporo's registry until April 5, 2022, contained information on all the confirmed COVID-19 patients in Sapporo from the beginning of the pandemic. The patients were recorded for their age, sex, test-positive date, onset, severity during the acute phase of infection, and other information. Additionally, the Sapporo Public Health Office had the vaccination status of residents maintained in the National Vaccine Record System. These datasets can be linked with each other and the data of the present study by unique keys.

Epidemiologic situation in Sapporo

The daily cases, cumulative incidence, and vaccination coverage for those aged 20 to 64 in Sapporo from September 2020 were visualized (Figure 1). The main circulating variants were the wildtype until February 2021, the Alpha variant from March to June 2021, the Delta variant from July to December 2021, and the Omicron variant from January 2022.

Until the study period, COVID-19 cases were classified as the notifiable disease surveillance so that people who were suspected to have COVID-19 were tested. Additionally, before the surge of the Omicron variant, an active epidemiologic investigation was conducted for every patient to identify close contacts of the confirmed patients.

Appendix Table 1. Number and percentage of each symptom in a case-control study of patients with post–COVID-19 condition, Sapporo, Japan*

	No. months after SARS-CoV-2–positive test					
Symptoms	2–3 mo.	4–6 mo.	7–9 mo.	10–12 mo.	13–18 mo.	Control group
Fatigue	423/3,661 (11.55)	159/3,607 (4.41)	143/3,482 (4.11)	139/2,373 (5.86)	71/994 (7.14)	194/3,672 (5.28)
Anosmia	288/3,681 (7.82)	199/3,627 (5.49)	270/3,501 (7.71)	110/2,387 (4.61)	76/995 (7.64)	11/3,672 (0.30)
Ageusia	264/3,677 (7.18)	152/3,623 (4.20)	173/3,497 (4.95)	80/2,384 (3.36)	51/996 (5.12)	13/3,672 (0.35)
Cough	227/3,666 (6.19)	65/3,612 (1.80)	31/3,486 (0.89)	33/2,379 (1.39)	18/990 (1.82)	90/3,672 (2.45)
Fever, temp. ≥37C°	215/3,650 (5.89)	16/3,596 (0.44)	20/3,471 (0.58)	12/2,363 (0.51)	7/985 (0.71)	10/3,672 (0.27)
Dyspnea	210/3,678 (5.71)	125/3,623 (3.45)	103/3,497 (2.95)	137/2,386 (5.74)	56/996 (5.62)	54/3,672 (1.47)
Alopecia	195/3,691 (5.28)	397/3,636 (10.92)	129/3,510 (3.68)	70/2,393 (2.93)	44/999 (4.40)	70/3,672 (1.91)
Headache	135/3,676 (3.67)	64/3,624 (1.77)	64/3,498 (1.83)	73/2,382 (3.06)	46/994 (4.63)	149/3,672 (4.06)
Poor appetite	114/3,680 (3.10)	22/3,625 (0.61)	19/3,499 (0.54)	15/2,383 (0.63)	5/993 (0.50)	14/3,672 (0.38)
Diminished ability to concentrate	109/3,686 (2.96)	101/3,631 (2.78)	119/3,505 (3.40)	116/2,391 (4.85)	71/997 (7.12)	118/3,672 (3.21)
Pharyngalgia	99/3,677 (2.69)	17/3,623 (0.47)	16/3,497 (0.46)	7/2,385 (0.29)	5/994 (0.50)	35/3,672 (0.95)
Muscle weakness	88/3,687 (2.39)	41/3,632 (1.13)	53/3,506 (1.51)	54/2,391 (2.26)	20/997 (2.01)	36/3,672 (0.98)
Chest pain	82/3,684 (2.23)	38/3,629 (1.05)	44/3,503 (1.26)	41/2,390 (1.72)	21/999 (2.10)	31/3,672 (0.84)
Arthralgia or joint swelling	78/3,683 (2.12)	10/3,628 (0.28)	20/3,502 (0.57)	30/2,389 (1.26)	9/996 (0.90)	92/3,672 (2.51)
Rhinorrhea	73/3,683 (1.98)	19/3,628 (0.52)	23/3,502 (0.66)	24/2,390 (1.00)	15/997 (1.50)	150/3,672 (4.08)
Brain fog	64/3,692 (1.73)	69/3,637 (1.90)	72/3,511 (2.05)	84/2,394 (3.51)	38/1,000 (3.80)	49/3,672 (1.33)
Myalgia	58/3,686 (1.57)	25/3,631 (0.69)	22/3,506 (0.63)	22/2,390 (0.92)	8/997 (0.80)	49/3,672 (1.33)
Sleep disturbance	51/3,687 (1.38)	51/3,632 (1.40)	92/3,506 (2.62)	88/2,391 (3.68)	44/997 (4.41)	206/3,672 (5.61)
Diarrhea	41/3,689 (1.11)	14/3,635 (0.39)	22/3,509 (0.63)	13/2,393 (0.54)	3/999 (0.30)	109/3,672 (2.97)
Abnormal menstruation	23/2,166 (1.06)	15/2,126 (0.71)	26/2,052 (1.27)	14/1,407 (1.00)	5/596 (0.84)	83/2,081 (3.99)
Nausea or vomiting	39/3,687 (1.06)	13/3,632 (0.36)	14/3,506 (0.40)	9/2,391 (0.38)	6/998 (0.60)	21/3,672 (0.57)
Night sweats	35/3,690 (0.95)	8/3,635 (0.22)	7/3,509 (0.20)	12/2,392 (0.50)	0/998 (0.00)	13/3,672 (0.35)
Pleuritic chest pain	35/3,693 (0.95)	9/3,638 (0.25)	7/3,512 (0.20)	14/2,395 (0.58)	3/999 (0.30)	4/3,672 (0.11)
Erectile dysfunction	10/1,528 (0.65)	7/1,513 (0.46)	18/1,461 (1.23)	19/989 (1.92)	11/404 (2.72)	30/1,591 (1.89)
Skin rash	22/3,691 (0.60)	22/3,636 (0.61)	27/3,510 (0.77)	25/2,394 (1.04)	16/998 (1.60)	120/3,672 (3.27)
Tingling	22/3,692 (0.60)	10/3,637 (0.27)	15/3,511 (0.43)	18/2,394 (0.75)	7/999 (0.70)	29/3,672 (0.79)
Abdominal pain	20/3,691 (0.54)	7/3,636 (0.19)	9/3,510 (0.26)	9/2,395 (0.38)	5/999 (0.50)	44/3,672 (1.20)
Eye symptoms	19/3,690 (0.51)	17/3,635 (0.47)	44/3,509 (1.25)	57/2,392 (2.38)	23/997 (2.31)	180/3,672 (4.90)
Tinnitus	19/3,693 (0.51)	20/3,638 (0.55)	39/3,512 (1.11)	51/2,395 (2.13)	35/999 (3.50)	139/3,672 (3.79)
Constipation	6/3,693 (0.16)	5/3,638 (0.14)	10/3,512 (0.28)	9/2,395 (0.38)	6/1,000 (0.60)	145/3,672 (3.95)
Pseudo-chilblains on toes	6/3,693 (0.16)	4/3,638 (0.11)	5/3,512 (0.14)	7/2,395 (0.29)	6/1,000 (0.60)	14/3,672 (0.38)

*Values indicate no. affirmative/no. responses (%).

Appendix Table 2. Adjusted odds ratios for each symptom in a case-control study of patients with post–COVID-19 condition, Sapporo, Japan*

Symptoms	2–3 mo.	4–6 mo.	7–9 mo.	10–12 mo.	13–18 mo.
Fatigue	2.36 (1.97–2.81)	0.84 (0.67–1.04)	0.77 (0.62–0.97)	1.12 (0.90–1.41)	1.38 (1.04–1.84)
Anosmia	27.34 (14.94–50.04)	19.33 (10.51–35.55)	27.66 (15.10–50.65)	15.89 (8.53–29.62)	27.74 (14.65–52.54)
Ageusia	21.33 (12.19–37.30)	12.27 (6.94–21.67)	14.58 (8.28–25.68)	9.77 (5.42–17.62)	15.49 (8.38–28.65)
Cough	2.65 (2.06–3.40)	0.74 (0.53–1.02)	0.37 (0.24–0.55)	0.57 (0.38–0.85)	0.75 (0.45–1.25)
Fever, temp. ≥37C°	23.11 (12.23–43.67)	1.60 (0.72–3.54)	2.04 (0.95–4.38)	1.89 (0.82–4.40)	2.52 (0.95–6.65)
Dyspnea	4.08 (3.01–5.52)	2.42 (1.75–3.35)	2.11 (1.51–2.94)	4.21 (3.06–5.80)	4.07 (2.78–5.96)
Alopecia	2.89 (2.19–3.82)	6.62 (5.10–8.59)	2.02 (1.50–2.71)	1.57 (1.12–2.20)	2.37 (1.61–3.48)
Headache	0.87 (0.69–1.11)	0.41 (0.30–0.55)	0.43 (0.32–0.58)	0.74 (0.55–0.98)	1.12 (0.79–1.57)
Poor appetite	8.54 (4.89–14.91)	1.56 (0.80–3.07)	1.38 (0.69–2.77)	1.67 (0.80–3.47)	1.31 (0.47–3.65)
Diminished ability to concentrate	0.91 (0.70–1.18)	0.87 (0.66–1.13)	1.06 (0.82–1.37)	1.55 (1.19–2.01)	2.31 (1.71–3.13)
Pharyngalgia	2.83 (1.92–4.17)	0.48 (0.27–0.86)	0.47 (0.26–0.85)	0.31 (0.14–0.69)	0.53 (0.21–1.35)
Muscle weakness	2.56 (1.73–3.78)	1.24 (0.79–1.95)	1.65 (1.08–2.53)	2.47 (1.61–3.79)	2.23 (1.28–3.88)
Chest pain	2.66 (1.75–4.03)	1.24 (0.77–1.99)	1.48 (0.93–2.35)	2.04 (1.28–3.27)	2.44 (1.39–4.27)
Arthralgia or joint swelling	0.88 (0.65–1.20)	0.12 (0.06–0.22)	0.24 (0.15–0.39)	0.51 (0.34–0.78)	0.37 (0.19–0.74)
Rhinorrhea	0.47 (0.35–0.62)	0.12 (0.08–0.20)	0.16 (0.10–0.24)	0.24 (0.15–0.37)	0.36 (0.21–0.62)
Brain fog	1.28 (0.88–1.87)	1.41 (0.97–2.04)	1.55 (1.07–2.23)	2.72 (1.90–3.88)	2.94 (1.91–4.52)
Myalgia	1.23 (0.84–1.81)	0.56 (0.35–0.91)	0.50 (0.30–0.82)	0.72 (0.43–1.19)	0.63 (0.30–1.35)
Sleep disturbance	0.24 (0.17–0.32)	0.24 (0.17–0.33)	0.46 (0.36–0.59)	0.64 (0.50–0.83)	0.77 (0.55–1.08)
Diarrhea	0.37 (0.26–0.53)	0.13 (0.07–0.22)	0.21 (0.13–0.33)	0.18 (0.10–0.32)	0.10 (0.03–0.32)
Abnormal menstruation	0.25 (0.16–0.40)	0.17 (0.09–0.29)	0.30 (0.19–0.47)	0.24 (0.13–0.42)	0.20 (0.08–0.50)
Nausea or vomiting	1.74 (1.02–2.98)	0.57 (0.29–1.15)	0.63 (0.32–1.25)	0.61 (0.28–1.34)	0.98 (0.39–2.45)
Night sweats	2.70 (1.43–5.13)	0.63 (0.26–1.52)	0.57 (0.22–1.42)	1.44 (0.65–3.16)	NA
Pleuritic chest pain	8.85 (3.14–24.94)	2.36 (0.72–7.68)	1.77 (0.52–6.08)	5.22 (1.71–15.90)	NA
Erectile dysfunction	0.36 (0.17–0.74)	0.25 (0.11–0.58)	0.68 (0.38–1.23)	1.04 (0.58–1.86)	1.53 (0.76–3.10)
Skin rash	0.18 (0.12–0.29)	0.19 (0.12–0.30)	0.24 (0.16–0.36)	0.32 (0.21–0.49)	0.49 (0.29–0.84)
Tingling	0.77 (0.44–1.34)	0.36 (0.17–0.73)	0.55 (0.29–1.03)	NA	0.93 (0.40–2.12)
Abdominal pain	0.43 (0.25–0.74)	0.16 (0.07–0.35)	0.21 (0.10–0.43)	0.30 (0.15–0.62)	0.40 (0.16–1.02)
Eye symptoms	0.10 (0.06–0.17)	0.09 (0.06–0.16)	0.26 (0.18–0.36)	0.49 (0.36–0.66)	0.47 (0.30–0.73)
Tinnitus	0.13 (0.08–0.22)	0.14 (0.09–0.23)	0.29 (0.20–0.42)	0.56 (0.41–0.78)	0.94 (0.64–1.37)
Constipation	0.04 (0.02–0.09)	0.03 (0.01–0.08)	0.07 (0.04–0.13)	0.09 (0.05–0.18)	0.14 (0.06–0.32)
Pseudo-chilblains on toes	0.43 (0.17–1.13)	0.29 (0.10–0.90)	0.38 (0.14–1.05)	0.78 (0.32–1.95)	1.63 (0.62–4.26)

*Timeframes represent months post illness onset among case-patients. Odds ratios are adjusted for age and sex and results from controls were used as a reference. NA, not applicable.

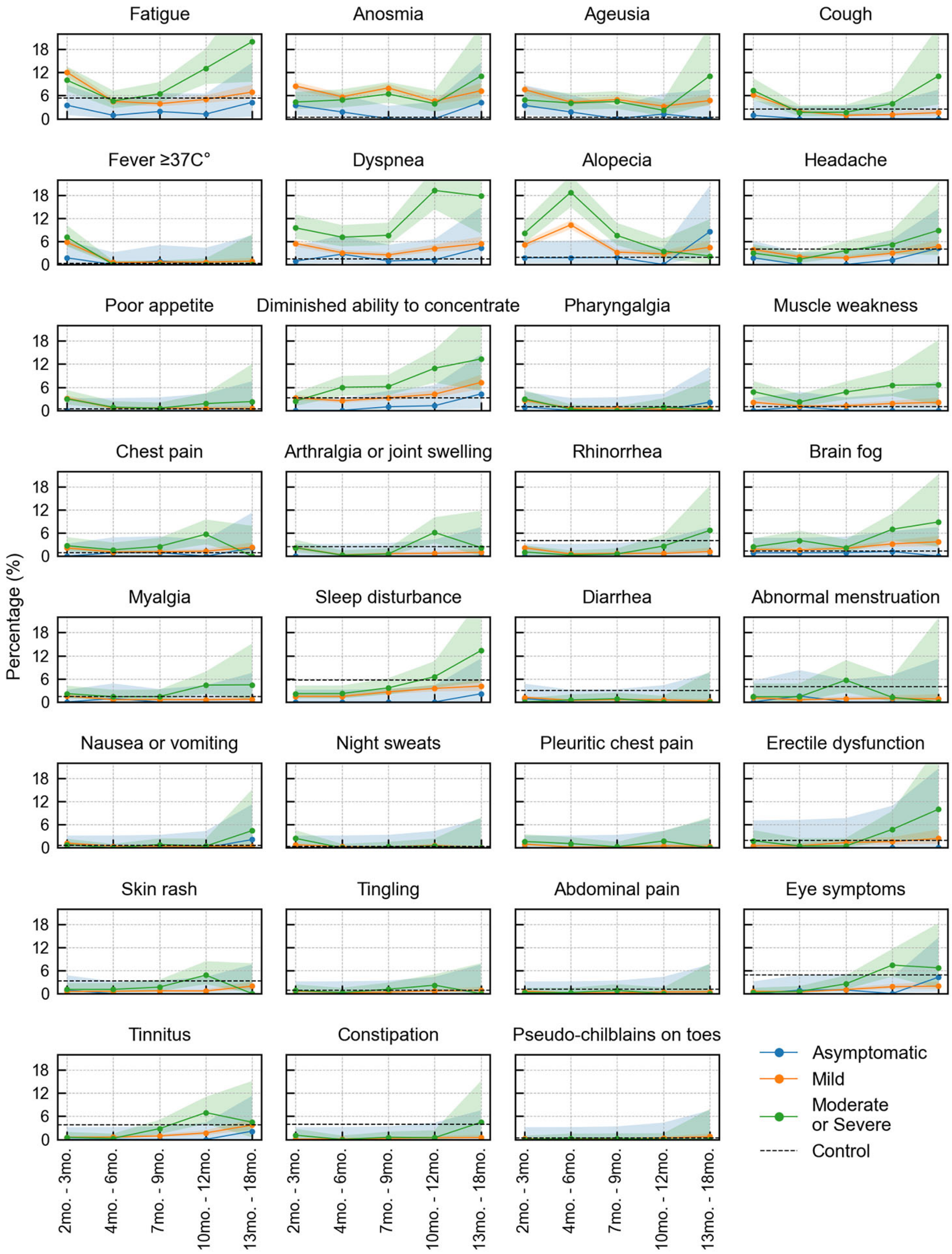

Appendix Figure 1. Line plots of the prevalence of each symptom in a case-control study of long COVID, Sapporo, Japan. Symptoms among COVID-19 case-patients are shown at designated timepoints from illness onset and stratified by severity compared with those of controls. Because the number of severe cases was small, moderate and severe cases were combined for visualization. Filled areas represent 95% CI.

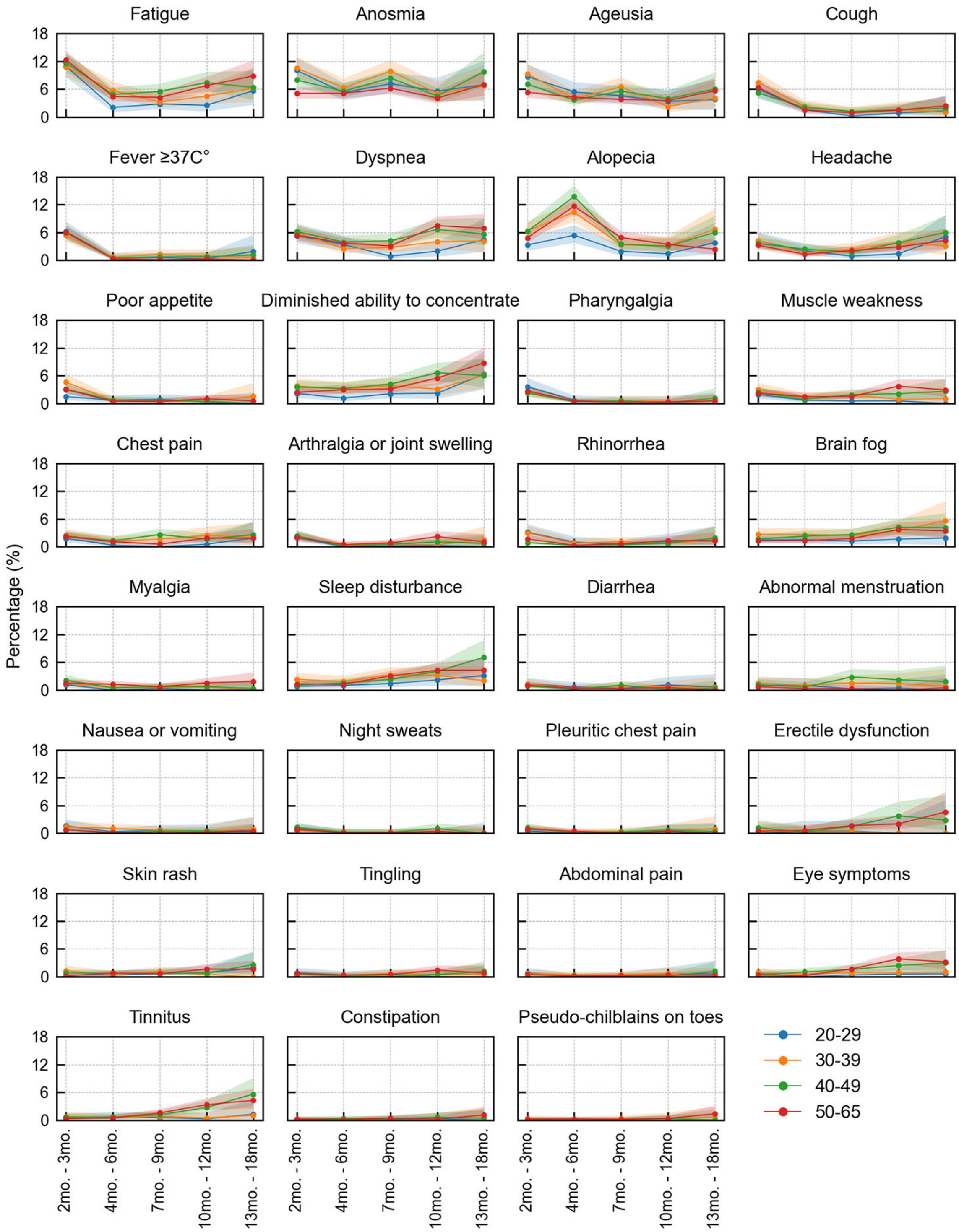

Appendix Figure 2. Line plots of the prevalence of each symptom in a case-control study of long COVID, Sapporo, Japan. Symptoms among COVID-19 case-patients are shown for each age range at designated timepoints from illness onset. Filled areas represent 95% CI.

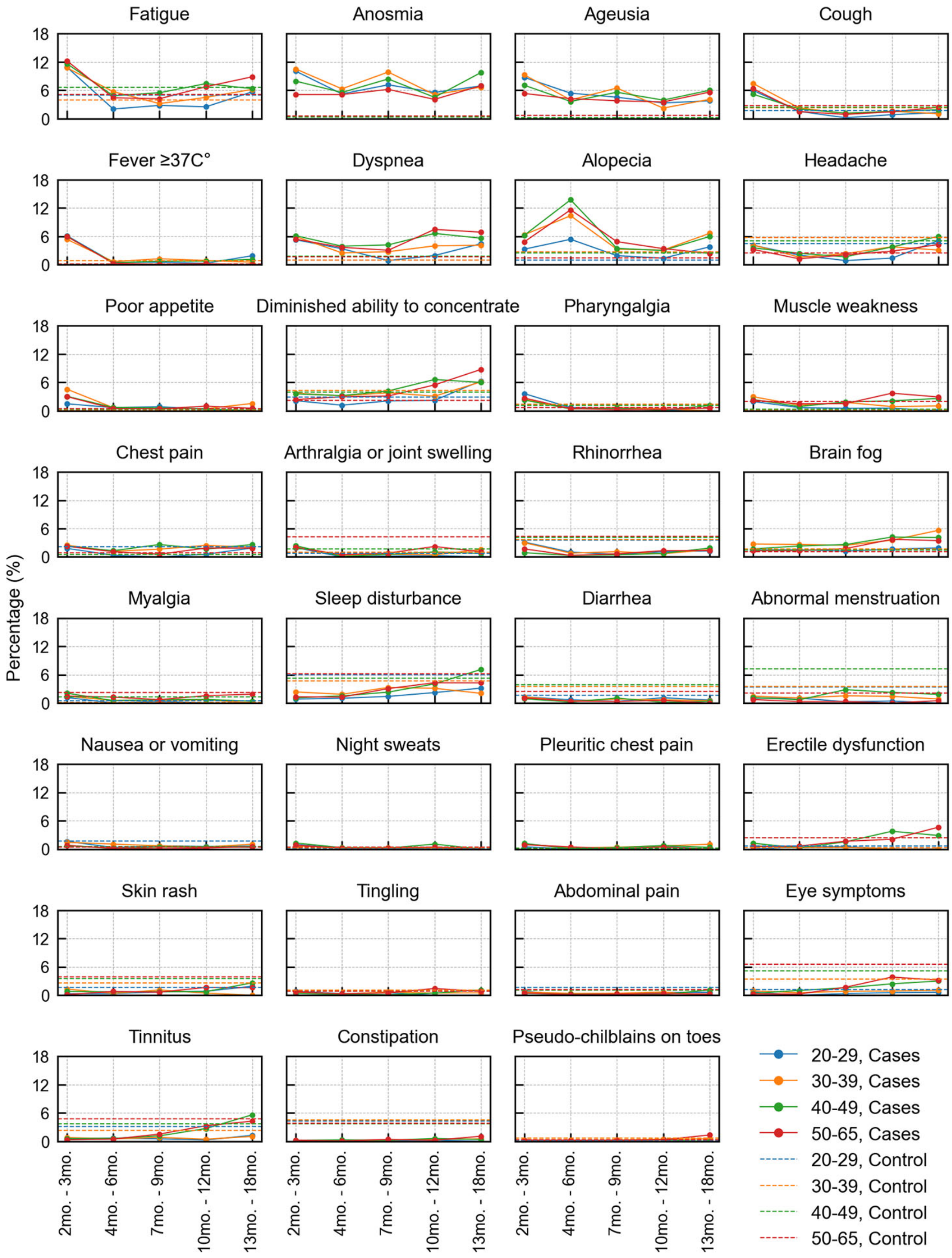

Appendix Figure 3. Line plots of the prevalence of each symptom in a case-control study of long COVID, Sapporo, Japan. Symptoms among COVID-19 case-patients are shown at designated timepoints from illness onset and controls are shown at time of responding to questionnaire. Filled areas represent 95% CI.

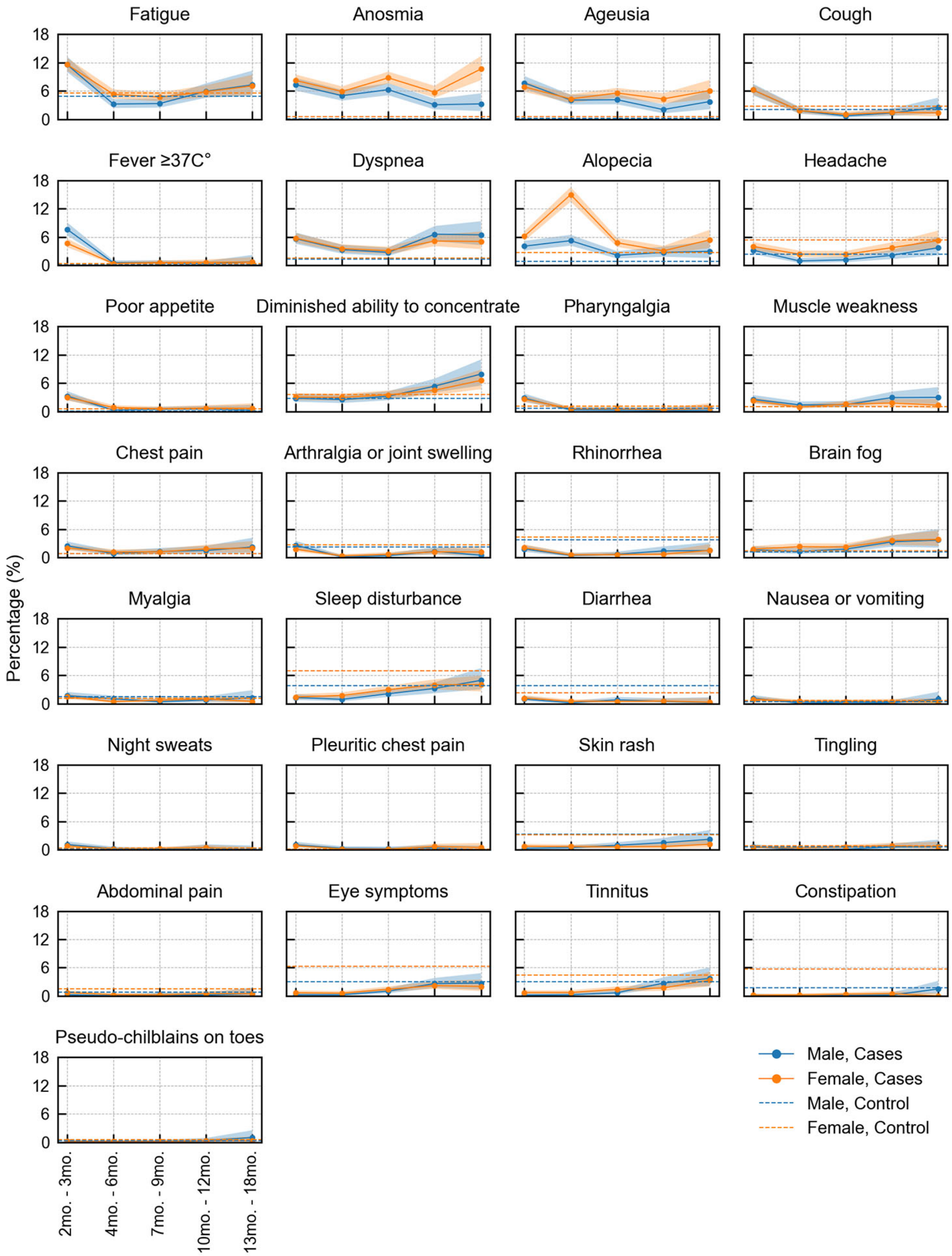

Appendix Figure 4. Line plots of the prevalence of each symptom in a case-control study of long COVID, Sapporo, Japan. Symptoms among COVID-19 male and female case-patients are shown at designated timepoints from illness onset and controls are shown at time of responding to questionnaire. Filled areas represent 95% CI.

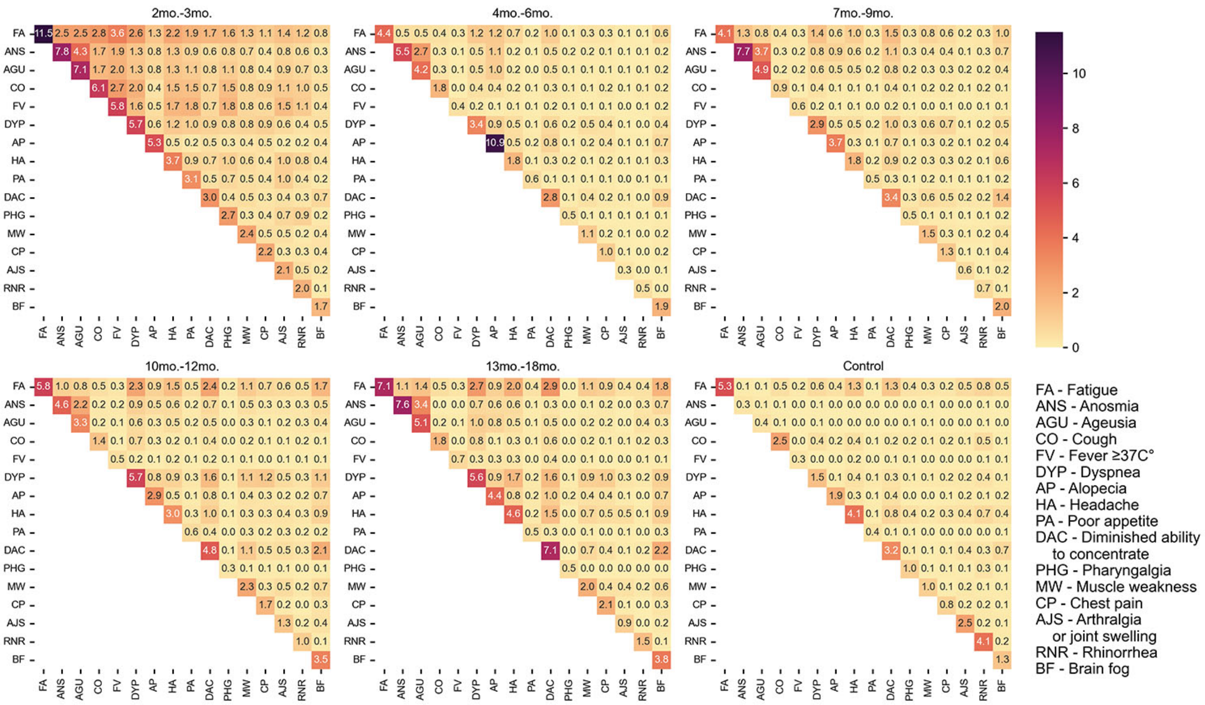

Appendix Figure 5. Heat map of prevalence of each symptom in a case-control study of long COVID, Sapporo, Japan. Symptoms among COVID-19 case-patients are shown at designated timepoints from illness onset and controls are shown at time of responding to questionnaire.
